# Ultra-Uniform and Very Thin Ag Nanowires Synthesized via the Synergy of Cl^−^, Br^−^ and Fe^3+^ for Transparent Conductive Films

**DOI:** 10.3390/nano10020237

**Published:** 2020-01-29

**Authors:** Xiao-Ming Wang, Long Chen, Enrico Sowade, Raul D. Rodriguez, Evgeniya Sheremet, Chun-Mei Yu, Reinhard R. Baumann, Jin-Ju Chen

**Affiliations:** 1 School of Materials and Energy, University of Electronic Science and Technology of China, Chengdu 610054, China; 2 Digital Printing and Imaging Technology, Chemnitz University of Technology, 09126 Chemnitz, Germany; 3 Research School of Chemistry and Applied Biomedical Sciences, Tomsk Polytechnic University, 30 Lenin Ave, Tomsk 634050, Russia; 4 Research School of Physics, Tomsk Polytechnic University, 30 Lenin Ave, Tomsk 634050, Russia

**Keywords:** silver nanowires, mediated agents, synergy, transparent conductive films, flexible electronics

## Abstract

The properties and applications of Ag nanowires (AgNWs) are closely related to their morphology and composition. Therefore, controlling the growth process of AgNWs is of great significance for technological applications and fundamental research. Here, silver nanowires (AgNWs) were synthesized via a typical polyol method with the synergistic effect of Cl^−^, Br^−^, and Fe^3+^ mediated agents. The synergistic impact of these mediated agents was investigated intensively, revealing that trace Fe^3+^ ions provided selective etching and hindered the strong etching effect from Cl^−^ and Br^−^ ions. Controlling this synergy allowed the obtainment of highly uniform AgNWs with sub-30 nm diameter and an aspect ratio of over 3000. Transparent conductive films (TCFs) based on these AgNWs without any post-treatment showed a very low sheet resistance of 4.7 Ω sq^−1^, a low haze of 1.08% at a high optical transmittance of 95.2% (at 550 nm), and a high figure of merit (FOM) of 1210. TCFs exhibited a robust electrical performance with almost unchanged resistance after 2500 bending cycles. These excellent high-performance characteristics demonstrate the enormous potential of our AgNWs in the field of flexible and transparent materials.

## 1. Introduction

With the development of nanotechnology, metal nanomaterials have been widely investigated due to their unique physical and chemical properties, especially one-dimensional silver nanowires (AgNWs) [1,2,3]. AgNWs are extensively used in surface-enhanced Raman scattering [4,5,6,7], transparent conductive films (TCFs) [8,9,10,11,12], sensors [13,14,15,16,17], electronic circuits [18,19,20,21,22], and other electronic devices owing to their excellent electrical, thermal, optical, and mechanical properties. The performance and corresponding applications of AgNWs are closely related to their size and microstructure [23,24]. Especially for use in TCFs, AgNWs are highly required with small diameter, high aspect ratio and uniformity [25,26,27].

At present, various methods for the preparation of AgNWs have been reported. Among them, the polyol thermal method is widely used due to its simple operation and friendly reaction environment [28,29,30]. Mediated agents (including anion or cation mediated agents) play an essential role in the synthesis of AgNWs by regulating the growth and etching speed [31]. Some reports [32,33,34,35,36,37,38,39] showed that anions such as Cl^−^ ions and Br^−^ ions could reduce supersaturation, thus promoting the formation of multiple twins and accelerating the growth of AgNWs. The etching effect of anion mediated agents was also investigated in detail. Wiley et al. [40] explored the effect of Cl^−^ ions and found that a high concentration of Cl^−^ ions could etch multiple twins under the action of oxygen atoms in the system. The same authors [41] also explored the effect of Br^−^ ions, finding that high concentrations of Br^−^ ions led to the conversion of multiple twins to single twins, which was not conducive to the synthesis of AgNWs. Some reports [41,42,43,44,45,46] confirmed that metal cations such as Cu^2+^ ions and Fe^3+^ ions could prevent the occurrence of an oxidative etching by consuming dissolved oxygen in the reaction system, thereby improving the quality of AgNWs. Similarly, cation mediated agents also exhibited the etching effect. Zhang et al. [1] explored the impact of FeCl_3_ on the morphology of AgNWs, considering that the oxidative etching of Fe^3+^ ions could reduce the diameter of AgNWs to a certain extent. Zhan et al. [31] studied the effect of Fe^3+^ ions by changing the Fe^3+^ ion concentration. They found that the oxidative etching of high concentration Fe^3+^ ions could reduce the number of seeds, leading to larger size AgNWs. Although the effects of anions and cations on the microstructure of AgNWs have been widely investigated, research on their synergistic effects on the growth of AgNWs remains mostly unexplored. This is an important question since it is expected that the AgNW microstructure can be regulated by the synergistic action of anion and cation mediated agents.

In our previous work [47], it was demonstrated that Cl^−^ ions can effectively reduce the diameter of AgNWs but will cause AgNWs to be uneven, and Fe^3+^ ions can improve the uniformity of AgNWs. In this work, AgNWs are synthesized via a typical polyol method using poly(N-vinylpyrrolidone) (PVP) as the capping agent. The synergistic effect of anions (Cl^−^ ions and Br^−^ ions) and cations (Fe^3+^ ions) on the microstructure of AgNWs is investigated in depth. We obtain highly uniform AgNWs with a very thin diameter (~29 nm) and an ultra-high aspect ratio (over 3000) by tuning the amount of different mediated agents. Based on these outstanding AgNWs, TCFs show excellent performance with a low sheet resistance (4.7 Ω sq^−1^), a low haze of 1.08% at high transmittance (95.2% at 550 nm), and a high figure of merit (FOM, 1210), while maintaining electrical conductivity after 2500 bending cycles.

## 2. Materials and Methods

### 2.1. Materials

All chemical reagents were analytical grade pure and could be used without further purification, purchased from Chengdu Kelong Chemical Co., Ltd. (Chengdu, China), including AgNO_3_, ethylene glycol (EG), poly(N-vinylpyrrolidone) (PVP-K90, *M*w ≈ 1300,000), NaCl, NaBr, Fe(NO_3_)_3_, FeCl_3_·6H_2_O and absolute ethanol (99.7 wt.%). The deionized water used had a conductivity of less than 0.055 μs/cm. Transparent conductive films were prepared with mixed cellulose esters (MCE) membranes with a pore size of 0.22 μm.

### 2.2. Synthesis of AgNWs and Preparation of TCFs

PVP dissolved in ethylene glycol with a concentration of 0.15 M was first prepared, and a certain amount of mediated agents was added to the solution. AgNO_3_ dissolved in ethylene glycol with a concentration of 0.1 M was also prepared. Then, the two solutions above were mixed in a volume ratio of 1:1. Afterward, the mixture was poured into a Teflon-lined stainless-steel autoclave and kept at 160 °C for a certain time. Finally, AgNW products were obtained after being rinsed with absolute ethanol three times.

TCFs were prepared by vacuum filtration. The as-prepared AgNWs were used as conductive components of TCFs, and MCE membrane acted as a support material. The AgNWs were dispersed into deionized water and evenly mixed under stirring (300 rpm) for 3 min. Then, the mixed solution was filtered through the MCE membrane. After drying at 40 °C, the sample was treated with acetone vapor for 30 s at 85 °C to obtain the free-standing TCFs. Detailed conditions for all products in this study are described in Supporting Information.

### 2.3. Characterization

The products’ morphology was observed by a scanning electron microscope (SEM, JEOL JSM-6490LV, Tokyo, Japan). The crystal structure of products was characterized by X-ray diffraction (XRD, Panalytical Empyrean, Almelo, the Netherlands). Ultraviolet-visible (UV-vis) absorption spectrum was recorded by an optical spectrometer (Agilent Cary 5000, Palo Alto, CA, USA). The structure of AgNWs was further confirmed by using a transmission electron microscope (TEM, JEOL-2100, Tokyo, Japan). The TCFs’ conductivity was tested by using a four-probe platform (Keithley RTS-2, Cleveland, Ohio, America). The chemical composition of AgNWs was analyzed using X-ray photoelectron spectroscopy (XPS, Phi-5000, UIvacPhi, Tokyo, Japan) and energy dispersive X-ray spectroscopy (EDS, Genesis 2000 XMS, Mahwah, NJ, USA). The morphology and surface roughness of TCFs were observed using atomic force microscopy (AFM, Park Systems Corp., Seoul, Korea).

## 3. Results and Discussion

**Figure 1:**
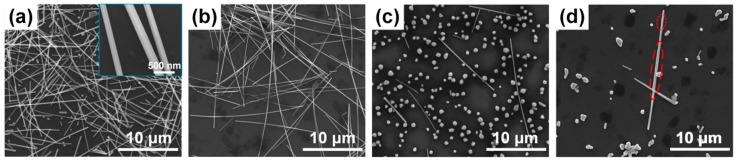
Scanning electron microscope (SEM) images of products synthesized with different concentrations of Fe(NO_3_)_3_: (**a**) 0 μM, (**b**) 10 μM, (**c**) 50 μM, and (**d**) 150 μM.

AgNWs were prepared by the polyol method without any mediated agents in the case of a ratio of PVP to AgNO_3_ of 1.5:1. This method produced AgNWs with a large diameter of about 200 nm and a yield of about 80%, as shown in Figure 1a. In the reaction system, a sufficient amount of PVP is preferentially adsorbed to the Ag (100) crystal plane, causing the crystal nucleus to grow along the Ag (111) plane [48], which is favorable for the formation of AgNWs. However, a large number of free silver ions are rapidly reduced by ethylene glycol, and the excessively fast reaction rate results in the formation of large-diameter and uneven AgNWs. Previous studies [1,31,40,41] showed that the AgNW morphology could be regulated by mediated agents. To explore the role of Fe^3+^ ions in the synthesis of nanosilver, we varied the concentration of Fe(NO_3_)_3_ from 10 to 150 μM. SEM images of these nanosilver samples are shown in Figure 1b–d. When the concentration of Fe^3+^ ions is low (Figure 1b), AgNWs with better uniformity and higher aspect ratio are obtained due to the effect of consuming oxygen and selective etching of Fe^3+^ ions. When the concentration of Fe^3+^ ions reaches 50 μM (Figure 1c), the yield of AgNWs is reduced to less than 10%. Multiple twin seeds, which will grow into AgNWs, are etched to dissolve before evolving into a stable silver wire due to the etching effect of Fe^3+^ ions [31], resulting in the formation of a large number of silver particles. As the concentration of Fe^3+^ ions increases, AgNWs gradually become thicker, and it can be clearly seen that the wire is uneven in diameter as marked in Figure 1d. The enhanced etching effect of Fe^3+^ ions leads to the dissolution of thinner silver wires. Dissolved Ag ions redeposit on the surface of other silver wires, resulting in the large and uneven diameter of AgNWs. Obviously, a trace amount of Fe^3+^ ions has already produced a certain degree of etching, and the etching effect greatly enhances with the increase in its concentration, strongly affecting the AgNW morphology.

**Figure 2:**
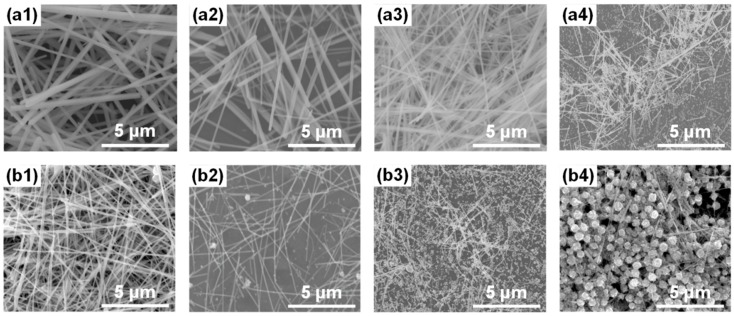
SEM images of products synthesized with different additions of (**a1**–**a4**) NaCl and (**b1**–**b4**) NaBr. The amount is (**a1**,**b1**) 150 μM, (**a2**,**b2**) 300 μM, (**a3**,**b3**) 600 μM, and (**a4**,**b4**) 900 μM, respectively.

Cl^−^ and Br^−^ were explored to regulate the structure of AgNWs by introducing NaCl and NaBr additives, respectively. In particular, the concentration of NaCl and NaBr varied from 150 to 900 μM. The corresponding SEM images of these AgNWs are shown in Figure 2(a1–a4,b1–b4). It can be seen that the concentration of anion mediated agents obviously affects the morphology of AgNWs. As shown in Figure 2(a1), almost exclusively AgNWs are formed at the Cl^−^ concentration of 150 μM. The concentration of free Ag^+^ ions in the solution decreased due to the formation of the AgCl colloid [49]. This colloid causes multiple twin seeds with lower surface energy to be preferentially formed. In addition, Cl^−^ ions form the Cl^−^/O_2_ pair with dissolved oxygen [33], which can selectively etch other seeds, increasing the AgNW yield. However, the concentration of free Ag^+^ ions quickly reduced by ethylene glycol is still high, resulting in the formation of AgNWs with a large diameter [47]. As the concentration of NaCl increases, the diameter of AgNWs gradually decreases, as shown in Figure 2(a2,a3). When the concentration of Cl^−^ ions reaches 600 μM, the morphology of AgNWs becomes more uniform and the diameter is reduced to about 100 nm. A large amount of silver chloride is formed due to the high concentration of Cl^−^ ions. This scenario not only decreases the formation rate of silver atoms but also provides a large number of nucleation sites for the generation of AgNWs, promoting the growth of small-diameter AgNWs [33]. However, when the Cl^−^ ion concentration increases to 900 μM (Figure 2(a4)), the large amount of AgCl colloids dramatically reduces the concentration of free Ag^+^ ions, limiting the growth of AgNWs. In addition, excessively high concentrations of Cl^−^ ions strengthen the etching of Cl^−^/O_2_, resulting in the dissolution of multiple twin seeds and the significant increase in silver particles [50,51,52,53,54]. Although AgNWs with a small diameter can be obtained at a Cl^−^ ion concentration of 600 μM, the diameter of AgNWs is still relatively large.

Compared with Cl^−^ ions, the Br^−^ ion has lower solubility of colloids in the order K_sp_(AgBr) < K_sp_(AgCl) << K_sp_(AgNO_3_) [47]. When a small amount of NaBr (150 μM) was used, almost no particles could be observed, as presented in Figure 2(b1). Obviously, the formation of AgNWs can be promoted by a low concentration of Br^−^ ions due to the selective etching of Br^−^/O_2_ [41], which is similar to the action of Cl^−^ ions. Significantly, the diameter of AgNWs is reduced to about 90 nm, which is better than the case of Cl^−^ ions with a concentration of 600 μM. The AgBr colloid with low solubility leads to less free Ag^+^ ions and more nucleation to grow into AgNWs with a smaller diameter [55]. As shown in Figure 2(b2), the diameter of AgNWs reduces to 58 nm at the Br^−^ concentration of 300 μM. However, a large number of particles appear in the product, which dramatically decreases the yield of AgNWs. In this case, the growth rate of multiple twin seeds is less than the etching speed, resulting in the dissolution of seeds before growing into AgNWs [56]. AgNWs are hardly generated as the concentration of Br^−^ ions further increases, as shown in Figure 2(b3). When the concentration of Br^−^ ions reaches 900 μM, the product almost completely consists of large particles, as shown in Figure 2(b4). The release rate of Ag^+^ ions is too slow at such a high concentration of Br^−^ ions, resulting in multiple twin seeds that grow into AgNWs being strongly etched by Br^−^/O_2_. The formed nanoparticles grow into large irregular particles under collision [35,57].

**Scheme 1:**
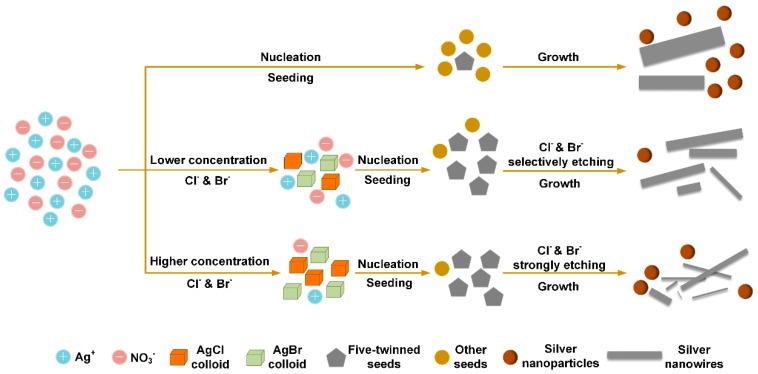
Schematic illustration of the synergistic effect of Cl^−^ and Br^−^ on the formation of silver nanowires (AgNWs).

**Figure 3:**
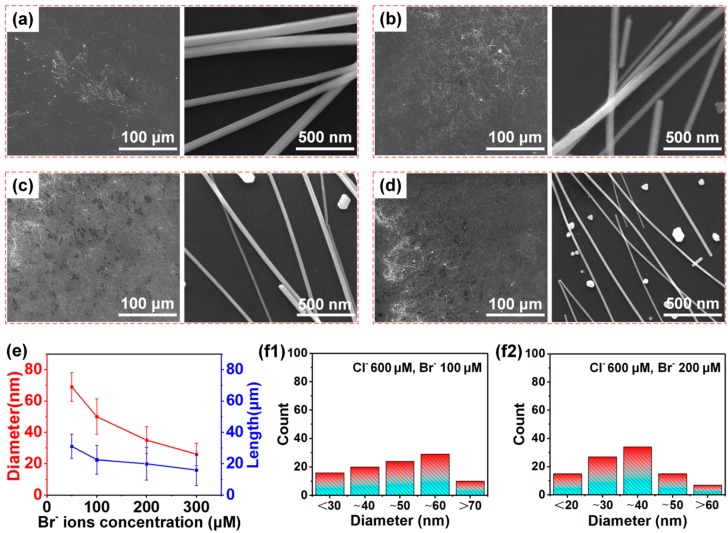
SEM images of products synthesized with 600 μM of NaCl and different additions of NaBr, which are (**a**) 50 μM, (**b**) 100 μM, (**c**) 200 μM, and (**d**) 300 μM, respectively. Changes in AgNW diameter and length with Br^−^ ion concentration are summarized in (**e**). The histograms of AgNW diameter are shown in (**f1**) and (**f2**) at 100 μM and 200 μM Br^−^ concentration, respectively.

It has been demonstrated that the diameter of AgNWs can be reduced more effectively by using Br^−^ than Cl^−^ ions, although silver nanoparticles are easily formed. Furthermore, AgNWs were synthesized with the simultaneous addition of NaCl and NaBr to establish the synergistic effect of Cl^−^ and Br^−^ ions on the morphology of AgNWs. The concentration of NaCl was fixed at 600 μM, while the concentration of NaBr varied from 50 to 300 μM. Scheme 1 shows the synergistic effect of Cl^−^ and Br^−^ mediated agents on the morphology of AgNWs. The morphologies of products observed by SEM are shown in Figure 3a–d. We observe that the diameter and the length distributions of AgNWs varied with the Br^−^ concentration, as illustrated in Figure 3e. The diameter distribution of AgNWs with 100 and 200 μM Br^−^ ions is shown in Figure 3(f1,f2), respectively. Results in Figure 3a–d show that the diameter of AgNWs significantly reduces compared to that with the addition of NaCl or NaBr alone. When the concentration of Br^−^ ions is 50 μM (Figure 3a), the diameter of AgNWs is 30 nm lower than that obtained when only using 600 μM NaCl (Figure 2(a3)). This result is attributed to the formation of insoluble AgBr colloids. Meanwhile, Cl^−^ and Br^−^ ions provide a certain degree of selective etching, as shown in Scheme 1. When the concentration of Br^−^ ions reaches a higher level of 100 μM (Figure 3b), the average diameter of AgNWs is reduced to 50 nm. A large number of AgCl and AgBr colloids are formed at the initial stage of the reaction, as shown in Scheme 1. These colloids slow down the rate of Ag^+^ ions being reduced and provide a large number of nucleation sites [33]. Therefore, this situation limits AgNW growth in the radial direction, producing samples with an ultra-small diameter [49]. However, many nanoparticles are generated due to the severe etching of Cl^−^/O_2_ and Br^−^/O_2_ to twin seeds and AgNWs. When the concentration of Br^−^ ions increases to 200 μM (Figure 3c), the average diameter of AgNWs can be further decreased to 35 nm. Afterwards, the further increment of Br^−^ ions leads to AgNWs with decreased diameter but also lower yield, as shown in Figure 3d.

It should be noted that the length of AgNWs synthesized by the synergy of NaCl and NaBr is shorter than 30 μm (Figure 3e) with a wide diameter distribution (Figure 3(f1,f2)). In addition, silver nanoparticles appear with decreasing diameter, resulting in a low yield of AgNWs. These observations can be explained as the strong etching of Cl^−^ ions and Br^−^ ions with dissolved oxygen in the reaction system [44]. It has been demonstrated in Figure 1 that Fe^3+^ ions can effectively regulate the morphology of AgNWs. The conversion between Fe^3+^ ions and Fe^2+^ ions can consume dissolved oxygen and effectively prevent the occurrence of oxidative etching [42], which is conducive to the growth of AgNWs. In this situation, Fe^3+^ ions can be reduced to Fe^2+^ ions by EG, and Fe^2+^ ions can also be oxidized to Fe^3+^ ions due to the presence of dissolved oxygen [58].

**Scheme 2:**
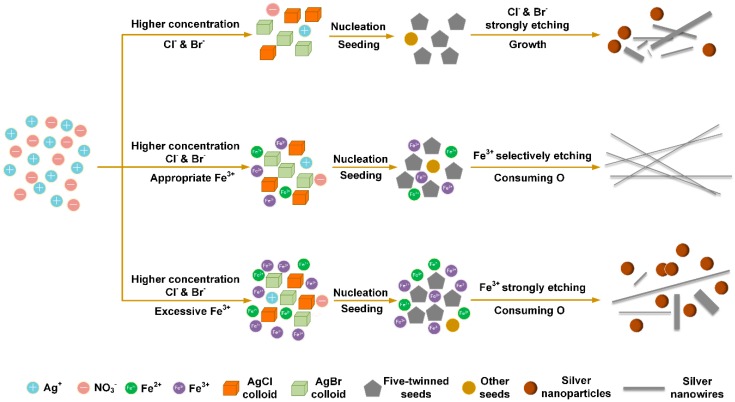
Schematic illustration of the synergistic effect of Cl^−^, Br^−^, and Fe^3+^ on the formation of AgNWs.

**Figure 4:**
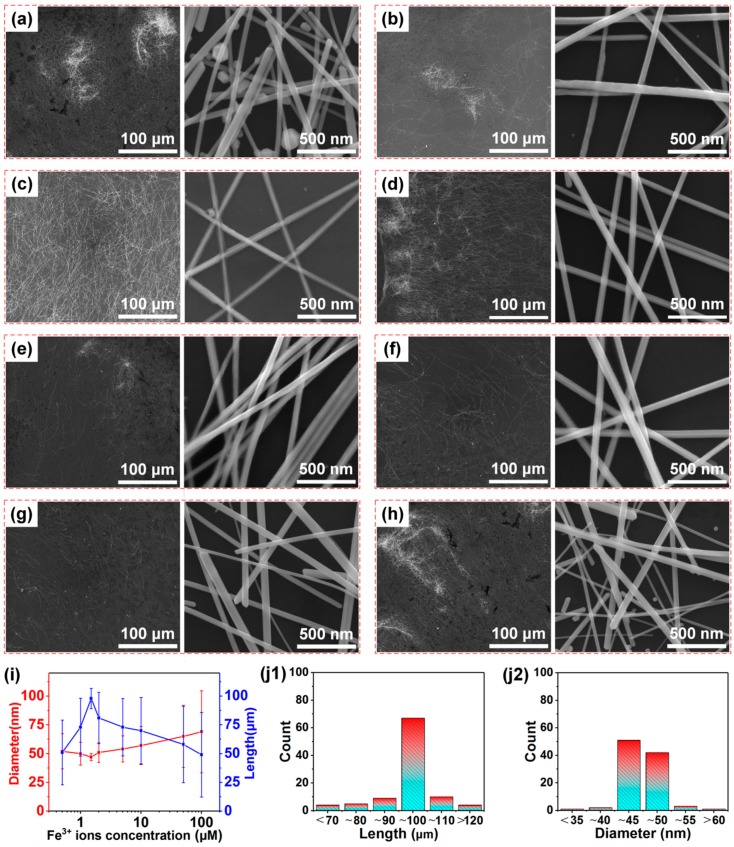
SEM images of products synthesized with 600 μM of NaCl, 100 μM of NaBr and different additions of Fe(NO_3_)_3_. The amount of Fe(NO_3_)_3_ is (**a**) 0.5 μM, (**b**) 1 μM, (**c**) 1.5 μM, (**d**) 2 μM, (**e**) 5 μM, (**f**) 10 μM, (**g**) 50 μM, and (**h**) 100 μM, respectively. Changes in AgNW diameter and length with Fe^3+^ ion concentration are summarized in (**i**). The histograms of AgNW length and diameter are, respectively, shown in (**j1**) and (**j2**) at 1.5 μM Fe^3+^ concentration.

Based on these results, the synergy of Cl^−^, Br^−^, and Fe^3+^ ions is investigated to control the morphology of AgNWs further. In this case, the concentration of NaCl and NaBr is fixed at 600 μM and 100 μM, respectively, and the concentration of Fe(NO_3_)_3_ varied from 0.5 to 100 μM. Scheme 2 shows the synergistic effect of Cl^−^, Br^−^ and Fe^3+^ mediated agents on the morphology of AgNWs. Corresponding SEM images of products are shown in Figure 4a–h, the diameter and length of AgNWs varying with Fe^3+^ ion concentration are illustrated in Figure 4i, and the length and diameter distributions of AgNWs for a Fe^3+^ concentration of 1.5 μM are shown in Figure 4(j1,j2), respectively. Compared to AgNWs synthesized without Fe^3+^ ions, the length of AgNWs significantly increases, as shown in Figure 4i, which fully demonstrates that Fe^3+^ ions play an important role in the growth of AgNWs. The average diameter of AgNWs gradually decreases at first and then increases when the concentration of Fe^3+^ ions increases. In particular, the AgNWs reach a maximum average length of 99 μm and a minimum average diameter of 47 nm when the concentration of Fe^3+^ ions is 1.5 μM.

When a small amount of Fe^3+^ ions (0.5 μM) is used, the average length of AgNWs increases to 50 μm (Figure 4a), and the average diameter reduces to 52 nm. However, some particles are still produced while AgNWs have a wide range of size distribution. Since Fe^3+^ ions at low concentration consume less oxygen, the quality of AgNWs improved only slightly due to the relatively strong etching effect of Cl^−^/O_2_ and Br^−^/O_2_. As the concentration of Fe^3+^ ions increases to 1 μM (Figure 4b), the quality of AgNWs is significantly improved. The average length of AgNWs reaches 70 μm, and the number of particles is further reduced. However, Fe^3+^ ions at this concentration are still insufficient to completely prevent the twin seed from being etched by Cl^−^/O_2_ and Br^−^/O_2_. When the concentration of Fe^3+^ ions is increased to an appropriate level of 1.5 μM, the uniformity of AgNWs is greatly improved with an average length of ~99 μm and an average diameter of ~47 nm. In addition, as shown in Figure 4c, the product is practically free of silver particles. Obviously, the aspect ratio of AgNWs is about 2000, which is critical for high-performance TCF applications. The length and diameter distributions of AgNWs displayed in Figure 4(j1,j2) further demonstrate that the uniformity of AgNWs is raised to an excellent level. As shown in Scheme 2, AgNWs with uniform morphology and high yield can be generated due to the following reasons. On the one hand, the oxidative etching that causes a severe unevenness of AgNWs is prevented since the dissolved oxygen atoms are completely consumed by the conversion between Fe^3+^ ions and Fe^2+^ ions [42]. On the other hand, multiple twin seeds that can grow fast into stabilized silver wires are not easily etched and other seeds are selectively etched into silver sources under this Fe^3+^ concentration, which leads to the great improvement in the length and yield of AgNWs. When the concentration of Fe^3+^ ions is further increased to 2 μM, the uniformity of AgNWs with a shorter average length of 70 μm and a larger average diameter of 51 nm becomes worse compared with the case of 1.5 μM, as shown in Figure 4d. At this concentration, the selective etching balance of Fe^3+^ ions is broken and partial twinned seeds are etched away. As the concentration of Fe^3+^ ions increases to higher levels of 5 μM (Figure 4e) and 10 μM (Figure 4f), the length and uniformity of AgNWs gradually decrease. This is due to the strong etching effect of excessive Fe^3+^ ions on twin seeds and AgNWs, which makes them convert into other silver structures [44], as shown in Scheme 2. When the concentration of Fe^3+^ ions reaches 50 μM (Figure 4g), the etching effect of Fe^3+^ ions becomes stronger, leading to wider AgNW diameter distribution and increased particle formation. When the concentration of Fe^3+^ ions further increases (Figure 4h), the uniformity of AgNWs becomes noticeably worse also with a lower yield. At this time, the capping function of PVP is destroyed due to the reaction between Fe^3+^ ions and the oxygen atoms of PVP [47]. This Fe^3+^-PVP reaction causes AgNWs to accelerate in the radial direction and become severely uneven. In general, controlling the etching effect of Fe^3+^ ions results in advantageous growth of AgNWs with high yield, high uniformity, and ultra-high aspect ratio. Notably, the suitable concentration of Fe^3+^ ions is very low relative to that of anion mediated agents.

**Figure 5:**
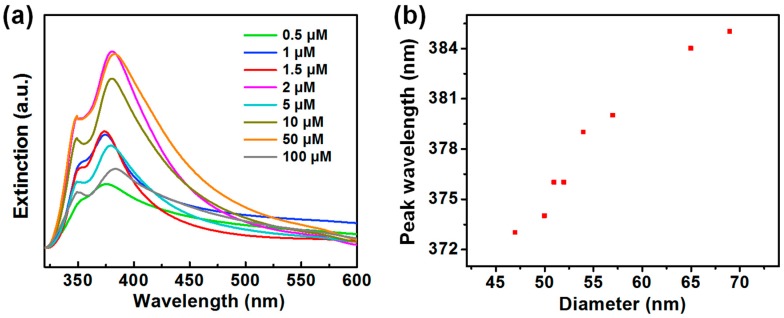
(**a**) UV-vis absorption spectra of products synthesized with 600 μM of NaCl, 100 μM of NaBr and different additions of Fe(NO_3_)_3_. (**b**) The quantitative statistics of the relationship between the strong absorption peak and AgNW diameter.

The UV-vis spectra of AgNWs synthesized with different concentrations of Fe^3+^ ions are shown in Figure 5a, and the corresponding shift of the strong extinction peak with the AgNW diameter is illustrated in Figure 5b. AgNWs can exhibit surface plasmon resonance bands due to the collective excitation of conduction-band electrons on the nanowire surface [44]. The weak peak appearing at about 350 nm is assigned to the quadrupole resonance excitation of AgNWs, and the strong peak located at about 380 nm is attributed to the transverse plasmon resonance of AgNWs, which has a high sensitivity to the dimension of AgNWs [31]. The absorption peak of silver nanoparticles at about 420 nm is absent in all samples, indicating that AgNWs are the main products. As the concentration of Fe^3+^ ions increases from 0.5 to 1.5 μM, the strong absorption peak blueshifts from 376 to 373 nm due to the smaller diameter of AgNWs with 1.5 μM Fe^3+^ ions. The gradual decrease in full width at half maximum (FHMW) is attributed to the AgNW yield increase. As the concentration of Fe^3+^ ions increases from 2 to 10 μM, the strong absorption peak gradually redshifts to 380 nm. As the concentration of Fe^3+^ ions further increases from 50 to 100 μM, the strong absorption peak exhibits a redshift to 385 nm. This redshift is accompanied by the gradual enlargement of FHMW due to the increasing diameter and the reduced yield of AgNWs. It can be seen in Figure 5b that the strong absorption peak increases approximately linearly with the increase in the diameter of AgNWs.

**Figure 6:**
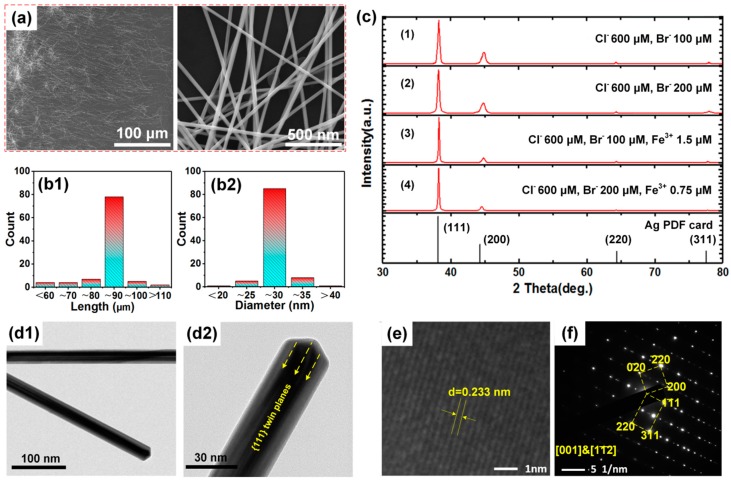
(**a**) SEM images of products synthesized with 600 μM of NaCl, 200 μM of NaBr and 0.75 μM of Fe(NO_3_)_3_. The histograms of length and diameter distribution of the AgNWs are, respectively, shown in (**b1**,**b2**). (**c**) X-ray diffraction (XRD) patterns of AgNWs prepared with different mediated agents. (**d1**,**d2**) Transmission electron microscope (TEM) images and (**e**) high-resolution TEM (HRTEM) image of the as-prepared AgNWs. (**f**) Selected area electron diffraction (SAED) pattern taken from an individual Ag nanowire.

Here, it has been demonstrated that uniform AgNWs with an aspect ratio of 2106 can be synthesized with 600 μM NaCl, 100 μM NaBr and 1.5 μM Fe(NO_3_)_3_. To achieve AgNWs with a higher aspect ratio, the Br^−^ mediated agent is adjusted to 200 μM due to its positive effect on diameter decreasing, and the Fe^3+^ ion concentration is adjusted to 0.75 μM. SEM images of the as-prepared AgNWs are shown in Figure 6a, and the length and diameter distributions of the AgNWs are, respectively, illustrated in Figure 6(b1,b2). As shown in Figure 6a, AgNWs show excellent uniformity and near 100% yield. Notably, the diameter of AgNWs is significantly reduced to ~29 nm, with a remarkably high length of ~90 μm. Thus, we obtain AgNWs with an ultra-high aspect ratio of about 3100. We verified the availability of this processing condition and further regulated the morphology of AgNWs. For longer reaction times, more AgNWs were obtained under the synergistic action of these three mediated agents. These results are shown in Figure S1 in Supporting Information.

XRD measurements were performed to analyze the crystal structure of typical products synthesized with the synergistic effect of mediated agents. Four diffraction peaks appearing in these diffraction spectra can be indexed as Ag (111), (200), (220), and (311) planes, which are consistent with the Ag crystal faced-centered cubic (FCC) phase (JCPDS file 04-0783), as shown in Figure 6c. The diffraction intensity ratios of (111) and (200) plane (I_111_/I_200_) of these four samples are much higher than the theoretical value as shown in Table S2, indicating that AgNWs grow along (111) facets and the growth along (100) facets is heavily limited due to capping by PVP [59]. However, some discrepancies exist in these samples owing to their different morphologies. The I_111_/I_200_ ratios of samples 1 and 2 are much lower than those from samples 3 and 4 synthesized with Fe^3+^ ions since the aspect ratios of the latter samples are much higher [60]. For samples 1 and 2, the FHMWs of the (111) diffraction peaks are much higher and the I_111_/I_220_ are significantly lower than those of samples 3 and 4, indicating that the crystallinity and yield of AgNWs prepared with Fe^3+^ ions is far higher. In contrast, the XRD patterns of AgNWs prepared with the addition of Cl^−^, Br^−^ or Fe^3+^, separately, are shown in Figure S2 in Supporting Information, and inferior performances are obviously observed in Table S2. In addition, XPS and EDS measurements were taken to analyze the composition of the AgNWs synthesized with Cl^−^, Br^−^ and Fe^3+^ mediated agents. Except for the weak C, O, and N signals from the PVP capping agent, Ag signal dominates in the XPS and EDS spectra as shown in Figure S3 in Supporting Information, and Cl, Br and Fe signals are not detected in the sample. The absence of mediated agents means that there is no residue of Cl^−^, Br^−^ and Fe^3+^ ions over the high-yield AgNWs after being thoroughly rinsed.

As discussed above, the AgNWs synthesized with 600 μM of NaCl, 200 μM of NaBr, and 0.75 μM of Fe(NO_3_)_3_ exhibit excellent microstructures. Here, high-resolution transmission electron microscopy (HRTEM) and selected area electron diffraction (SAED) were applied to investigate the AgNW crystal structure further. TEM images shown in Figure 6(d1,d2) show a highly consistent morphology of the AgNWs with SEM imaging results, with the {111} twin planes orienting parallel to the longitudinal axis. The HRTEM image shown in Figure 6e indicates that each portion of this twinned nanowire crystallizes well after growth. The lattice spacing is measured to be 0.233 nm, corresponding to the (111) facets of Ag crystal. Figure 6f shows the SAED of the AgNWs. The clear diffraction spots correspond to two sets of face-centered cubic lattices [61,62], and the corresponding twin axes are [001] and [1$\bar{1}$$\bar{2}$], respectively, also indicating the high crystallinity of the as-synthesized AgNWs.

**Figure 7:**
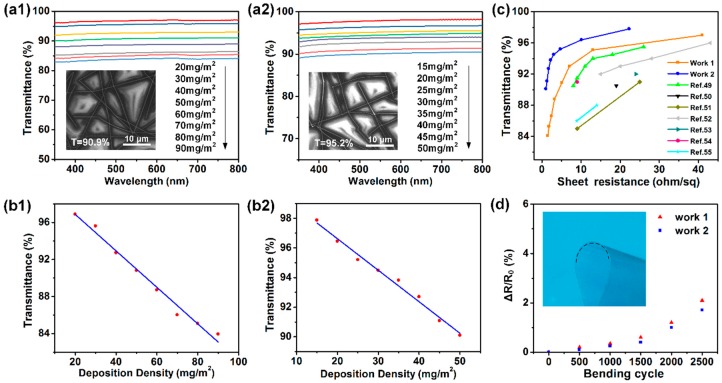
The transmittance of transparent conductive films (TCFs) varied with AgNW deposition density in (**a1**) work 1 and (**a2**) work 2. The transmittance of TCFs at 550 nm varied with deposition density in (**b1**) work 1 and (**b1**) work 2. (**c**) Transmittance versus sheet resistance for TCFs of our work and other reported results. (**d**) The resistance change of the TCFs after being subjected to repeated bending. Insets in Figure a1, a2 are SEM images of TCFs with optical transmittance of 90.9% and 95.2%, respectively. The inset in Figure d shows the bending angle of 180°.

TCFs based on AgNWs are considered to be the most promising materials to replace indium tin oxide for transparent display applications [26,27], and their performance is demonstrated to be closely related to the morphology and the aspect ratio of AgNWs [9,10,31]. AgNWs 1 with a diameter of ~29 nm and a length of ~90 μm, and AgNWs 2 with a diameter of ~47 nm and a length of ~99 μm were selected to prepare TCFs. Here, we label these samples as work 1 and work 2, respectively. The relationship between transmittance and AgNW deposition density is shown in Figure 7(a1) (work 1) and Figure 7(a2) (work 2) (the insets are SEM images of TCFs with optical transmittance of 90.9% and 95.2%, respectively), and the corresponding transmittance at 550 nm varied with deposition density, as respectively shown in Figure 7(b1,b2). The refractive index data of work 1 and work 2 are shown in Table S3 in Supporting Information. The relationship between square resistance and transmittance at 550 nm is depicted in Figure 7c, and the resistance change with bending cycles is shown in Figure 7d. The transmittance gradually decreases with increasing AgNWs deposition density in Figure 7(a1,a2). Correspondingly, the transmittance at 550 nm exhibits a linear correlation with deposition density in Figure 7(b1,b2). The transmittance at 550 nm is 90.9% with a deposition density of 50 mg/m^2^ for work 1, and it is up to 95.2% with a deposition density of 25 mg/m^2^ for work 2. It can be seen that the AgNWs are intertwined into a completely conductive network, as shown in the insets of Figure 7(a1,a2).

According to previous works [23,24], the transmittance of AgNW networks is usually described as:

(1) $$T_{opt}=T_{opt}^{0}(1-\alpha \cdot D_{dep})$$

where $T_{opt}^{0}$ is the substrate transmittance, $D_{dep}$ is the deposition density of AgNWs (in mg/m^2^), and α is the slope of the curve $T_{opt}$ versus $D_{dep}$. The slope is equal to:

(2) $$\alpha =\frac{4}{\pi d_{0}D_{0}}\cdot \left(1-\left(1-R_{opt}^{AgNW}\right)\cdot T_{opt}^{AgNW}\right)$$

where $R_{opt}^{AgNW}$ is the averaged optical reflection and $T_{opt}^{AgNW}$ is the averaged optical transmittance. Since the result in parentheses is close to unity [24], the prefactor $\frac{4}{\pi d_{0}D_{0}}$ is considered to be the determining parameter of α (here, $d_{0}$ is the density of bulk silver, and $D_{0}$ is the diameter of AgNWs). The slopes observed in Figure 7(b1) (17.1 × 10^−4^ m^2^/mg) and b2 (21.6 × 10^−4^ m^2^/mg) show the same trend as calculated values (26.1 × 10^−4^ m^2^/mg and 40.7 × 10^−4^ m^2^/mg), that is, the slope increases with a smaller diameter.

The figure of merit (FOM, defined as the electrical to optical conductivity ratio) is an important criterion for evaluating TCF performance [63,64,65]. Generally, FOM can be expressed as:

(3) $$T_{opt}={\left(1+\frac{Z_{0}}{2R_{s}}\cdot \frac{\sigma_{opt}}{\sigma_{dc}}\right)}^{-2}$$

where $\sigma_{dc}$ is the electrical conductivity, $\sigma_{opt}$ is the optical conductivity, and $Z_{0}$ is the impedance of free space (377 Ω). High values of FOM correspond to high transmittance coupled with low sheet resistance (*R_s_*) [62].

As shown in Figure 7c, the electrical conductivity of TCFs continues to improve while the transmittance decreases with AgNW deposition density increasing. For work 1, the *Rs* is 41.1 Ω sq^−1^ at a transmittance of 96.9%, meaning that a successful conductive path has formed. The *R_s_* presents a low value of 5 Ω sq^−1^ when the transmittance is 90.9% with a FOM of 771 for work 1. For work 2, the *R_s_* is as low as 4.7 Ω sq^−1^ at a high transmittance of 95.2% with a FOM of 1210, which is superior to TCF performances previously reported in other works [24,47,62,66,67,68,69,70,71]. Undoubtedly, work 2 based on AgNWs with a thinner diameter and a shorter length has better performance compared with work 1, which can be attributed to the diameter-dependent extinction efficiency of AgNWs [23]. This can also be confirmed by the haze characterization, as shown in Figure S4 in Supporting Information. Work 2 based on thinner AgNWs exhibits a lower haze index than work 1. A haze as low as 1.08% at 95.2% transmittance is obtained for work 2, indicating an excellent optical property. All these results show that the TCFs based on our AgNWs have excellent potential in applications as transparent electrodes. The high uniform morphology, small diameter, and ultra-high aspect ratio of the AgNWs all contribute to the outstanding performance of as-prepared TCFs. In addition, our TCFs exhibited retention of electrical performance after being bent for 2500 cycles (the inset shows the bending angle of 180°). As shown in Figure 7d, there is only a resistance change of about 2% after multiple bending cycles. Compared with work 1, work 2 shows better bending performance due to its higher aspect ratio.

**Figure 8:**
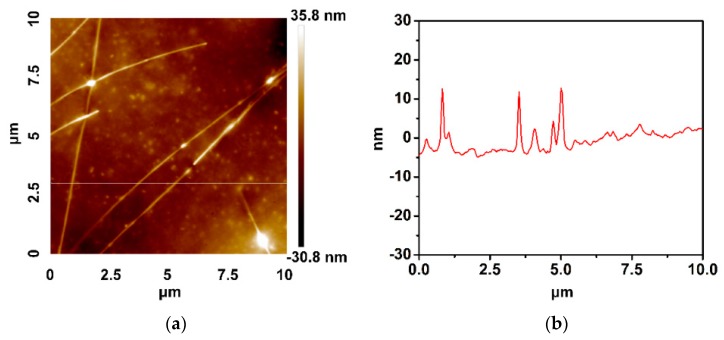
(**a**) Atomic force microscopy (AFM) image and (**b**) a scanned curve of the AgNW TCFs of work 2.

Atomic force microscopy (AFM) analysis was used to study the surface morphology of the fabricated TCFs. Figure 8 depicts an AFM image and a scanned curve for work 2, respectively. It can be clearly seen that the film is very even with a root mean square roughness (Rq) of 8.17 nm, which is an outstanding feature compared with other results of TCFs [9,12,25,71,72,73,74,75], and a comparison among them is described in detail in Table S4 in Supporting Information. Such a low surface roughness suggests that our method is able to build a nearly flat AgNW TCF.

## 4. Conclusions

The effect of Cl^−^, Br^−^, and Fe^3+^ ions on the morphology of AgNWs was investigated intensively. Cl^−^ ions are beneficial to the formation of AgNWs, Br^−^ ions can effectively reduce the diameter of AgNWs, and Fe^3+^ ions can resume the dissolved oxygen to balance the etching. Based on these effects, the morphology of AgNWs was controllably regulated under the synergistic action of Cl^−^, Br^−^, and Fe^3+^ mediated agents. In this way, highly uniform AgNWs with a small diameter of 29 nm and a high aspect ratio of 3100 were successfully obtained. With device applications in mind, TCFs based on the AgNWs showed superior optical and electrical performance, with a low sheet resistance of 4.7 Ω sq^−1^, a low haze of 1.08% at a transmittance of 95.2% at 550 nm, and a low roughness of 8.17 nm. The reliability and robustness of these devices were demonstrated by the excellent retention of electrical performance after 2500 bending cycles. These results imply that the synthesized AgNWs can offer great potential for many applications, especially for TCFs and wearable electronics.

## Supplementary Materials

The following are available online at https://www.mdpi.com/2079-4991/10/2/237/s1, Figure S1: SEM images of products synthesized with 600 μM of NaCl, 200 μM of NaBr and different additions of Fe(NO_3_)_3_. The amount of Fe(NO_3_)_3_ is (a) 0.5 μM, (b) 0.75 μM, (c) 1 μM, (d) 2 μM, (e) 5 μM, and (f) 10 μM, respectively. Changes in AgNW diameter with Fe^3+^ ion concentration are described in (g), and changes in AgNW length with Fe^3+^ ion concentration are described in (h). Figure S2: XRD patterns of AgNWs prepared with different mediated agents: (5) with only 600 μM NaCl, (6) with only 300 μM NaBr, and (7) with only 10 μM Fe(NO_3_)_3_. Figure S3: (a) XPS spectrum and (b) EDS analysis of as-prepared AgNWs. Figure S4: Haze of AgNW TCFs varied with transmittance. Table S1: Detailed conditions of all experiments in this study. Table S2: XRD analysis of AgNWs prepared with different mediated agents. Table S3: Refractive index data of transparent conductive films with different deposition densities at 374 nm. Table S4: Comparison of our AgNW TCFs with other similar kinds of work.
