# Supplementary material for: Ultra-Uniform and Very Thin Ag Nanowires Synthesized via the Synergy of Cl−, Br− and Fe3+ for Transparent Conductive Films
Source: Nanomaterials (Basel). 2020 Jan 29;10(2):237. doi: 10.3390/nano10020237 (PMC7075136; doi:10.3390/nano10020237)
Supplement: Supplementary file 1 [file nanomaterials-10-00237-s001.pdf]

Supporting Information

Ultra-uniform and very thin Ag nanowires synthesized *via* the synergy of Cl^- & Br^- & Fe^{3+} for transparent conductive films

Xiao-Ming Wang,[1] Long Chen,[1] Enrico Sowade,[2] Raul D. Rodriguez,[3] Evgeniya Sheremet,[4] Chun-Mei Yu,[1] Reinhard R. Baumann,[2] Jin-Ju Chen*[1]

[1] School of Materials and Energy, University of Electronic Science and Technology of China, Chengdu 610054, PR China

[2] Digital Printing and Imaging Technology, Chemnitz University of Technology, Chemnitz 09126, Germany

[3] Research School of Chemistry and Applied Biomedical Sciences, Tomsk Polytechnic University, 30 Lenin Ave, Tomsk 634050, Russia

[4] Research School of Physics, Tomsk Polytechnic University, 30 Lenin Ave, Tomsk 634050, Russia

Corresponding author:
Prof. Jin-Ju Chen

Tel: +86 28 83207590, Fax: +86 28 83202569,

E-mail address: jinjuchen@uestc.edu.cn

Table S1 Detailed conditions of all experiments in this study.

Samples	NaCl (μM)	NaBr (μM)	$Fe(NO_3)_3$ (μM)	Reaction time (min)
Fig. 1a	0	0	0	120
Fig. 1b	0	0	10	120
Fig. 1c	0	0	50	120
Fig. 1d	0	0	150	120
Fig. 2a1	150	0	0	120
Fig. 2a2	300	0	0	120
Fig. 2a3	600	0	0	120
Fig. 2a4	900	0	0	120
Fig. 2b1	0	150	0	120
Fig. 2b2	0	300	0	120
Fig. 2b3	0	600	0	120
Fig. 2b4	0	900	0	120
Fig. 3a	600	50	0	120
Fig. 3b	600	100	0	120
Fig. 3c	600	200	0	120
Fig. 3d	600	300	0	120
Fig. 4a	600	100	0.5	120

Fig. 4b	600	100	1	120
Fig. 4c	600	100	1.5	120
Fig. 4d	600	100	2	120
Fig. 4e	600	100	5	120
Fig. 4f	600	100	10	120
Fig. 4g	600	100	50	120
Fig. 4h	600	100	100	120
Fig. 6a	600	200	0.75	120
Fig. S1a	600	200	0.5	540
Fig. S1b	600	200	0.75	540
Fig. S1c	600	200	1	540
Fig. S1d	600	200	2	540
Fig. S1e	600	200	5	540
Fig. S1f	600	200	10	540

In order to further regulate the morphology of AgNWs, AgNWs were synthesized under the synergistic action of Cl^-, Br^-, and Fe^{3+} mediated agents at prolonging reaction time. Especially, the reaction time is increased from 2 h to 9 h under the conditions of 600 μM NaCl, 100 μM NaBr, and $Fe(NO_3)_3$ varied from 0.5 μM to 10 μM. Fig. S1a-S1f depict the SEM images of as-prepared products, and diameter and length of AgNWs varied with Fe^{3+} ions concentration are described in Fig. S1g and Fig. S1h, respectively. It is obviously shown that AgNWs with an average length of ~131 μm and an average diameter of ~55 nm are obtained at Fe^{3+} ions concentration of 0.75 μM. Since the increase in the usage of Br^- ions causing more AgBr colloid generation which could further reduce the reaction rate, a smaller amount of Fe^{3+} ions can achieve the optimal etching effect compared with that in Fig. 4. And ultra-uniform AgNWs with sub 30 nm diameter was successfully synthesized under this condition as shown in Fig. 6.

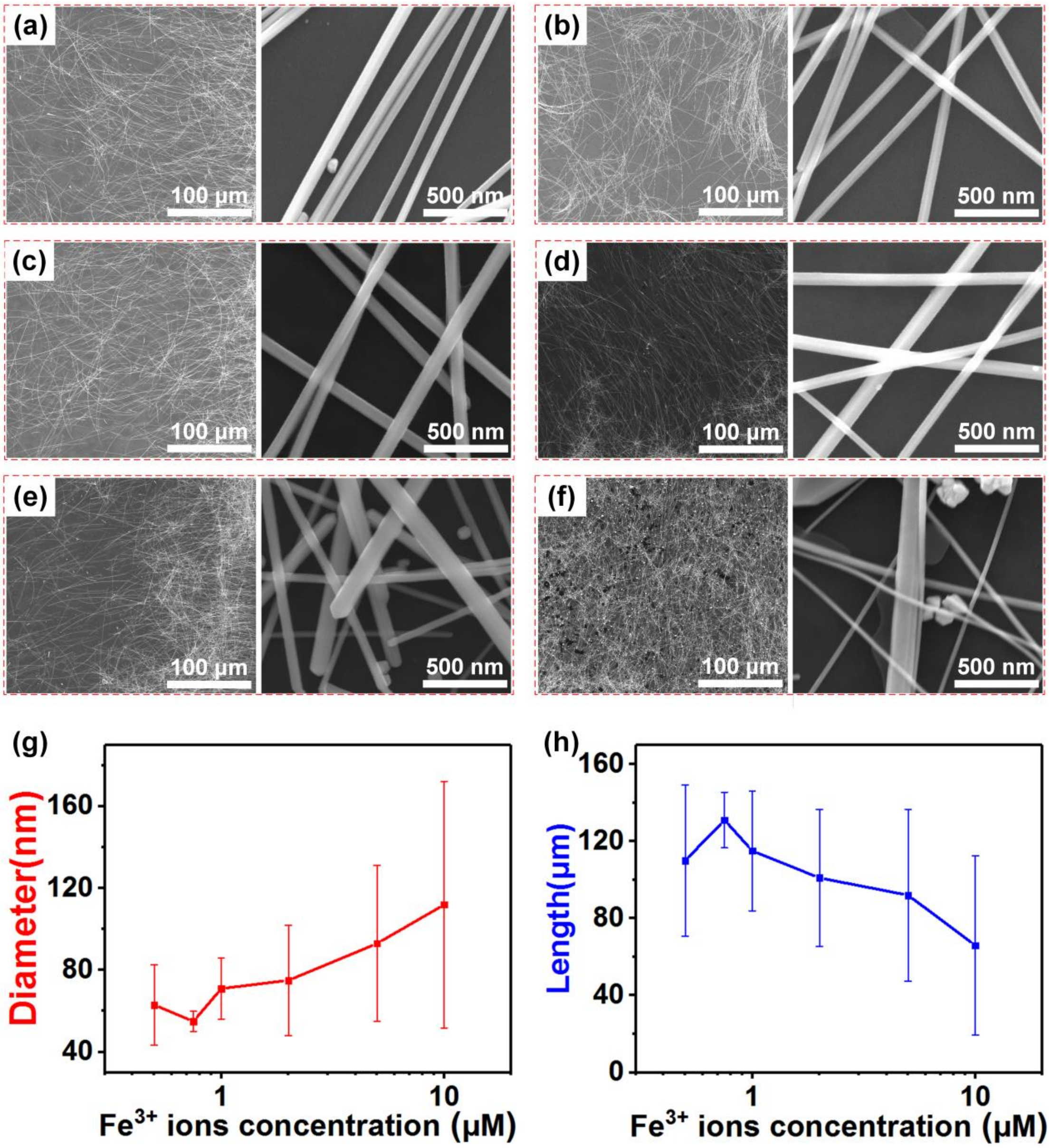

Figure S1. SEM images of products synthesized with NaCl of 600 μM, NaBr of 200 μM and different addition of $Fe(NO_3)_3$. The amount of $Fe(NO_3)_3$ is (a) 0.5 μM, (b) 0.75 μM, (c) 1 μM, (d) 2 μM, (e) 5 μM, (f) 10 μM, respectively. Changes in AgNWs diameter with Fe^{3+} ions concentration is described in (g), and changes in AgNWs length with Fe^{3+} ions concentration is described in (h).

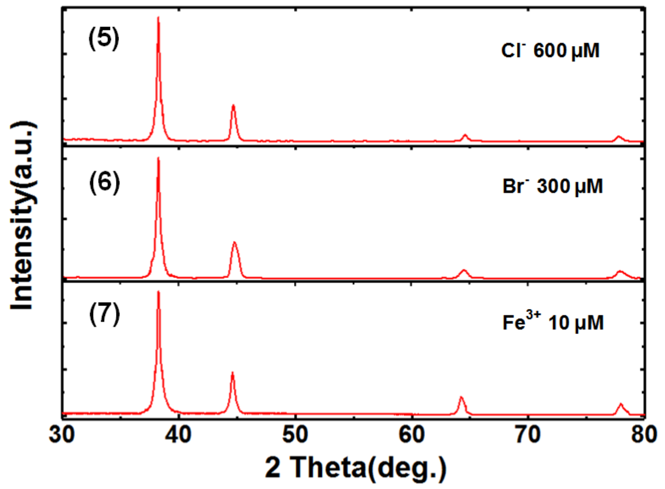

Figure S2. XRD patterns of AgNWs prepared with different mediated agents. (5) with only 600 μM NaCl, (6) with only 300 μM NaBr, and (7) with only 10 μM $Fe(NO_3)_3$.

Table S2 XRD analysis of AgNWs prepared with different mediated agents.

**Source**	**Aspect ratio**	**Yield of AgNWs(%)**	**I_{111}/I_{200}**	**I_{111}/I_{220}**	**FWHM of (111) diffraction peak**
JCPDS file 04-0783	/	/	2.5	4	/
Sample 1	450	61	3.58	19.84	0.31
Sample 2	571	38	4.01	17.89	0.34
Sample 3	2106	~100	9.25	72.76	0.171
Sample 4	3103	~100	10.19	67.03	0.176
Sample 5	153	~100	3.19	15.02	0.24
Sample 6	251	81	3.29	13.58	0.35
Sample 7	120	~100	2.86	6.49	0.30

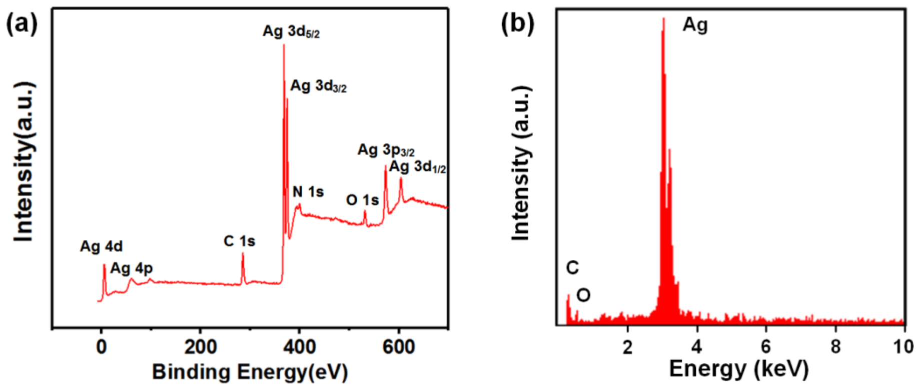

Figure S3. (a) XPS spectrum and (b) EDS analysis of as-prepared AgNWs.

Table S3 Refractive index data of transparent conductive films with different deposition densities at 374nm.

**Source**	**deposition density (mg/m^2)**	**Transmittance (%)**	**Refractive index**
Work 1	20	96.12	1.5876
Work 1	30	94.89	1.6149
Work 1	40	92.07	1.6781
Work 1	50	90.04	1.7241
Work 1	60	88.06	1.7698
Work 1	70	85.22	1.8367
Work 1	80	84.11	1.8634
Work 1	90	82.95	1.8917
Work 2	15	97.23	1.5385
Work 2	20	95.86	1.5684
Work 2	25	94.61	1.5958
Work 2	30	93.86	1.6123
Work 2	35	93,15	1.6279
Work 2	40	92.01	1.6532

Work 2	45	90.36	1.6901
Work 2	50	89.35	1.7127

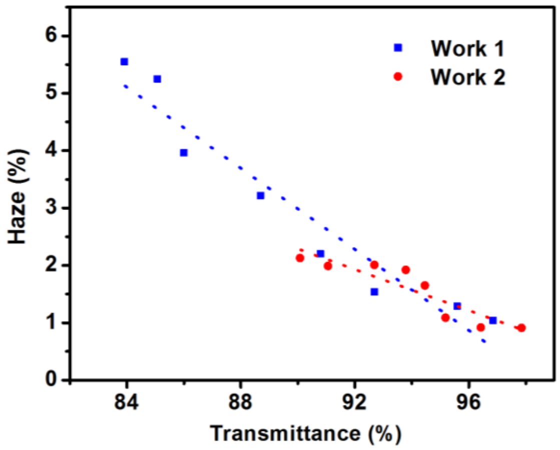

Figure S4. Haze of AgNWs TCFs varied with transmittance.

Work	Transmittance (%)	Sheet resistance (Ω sq^{-1})	Roughness (nm)
Our work	95.2	4.7	8.17
Ref. 9	91.8	14.3	14.7
Ref. 12	85.0	26.4	16.1
Ref. 24	89.2	2.9	-
Ref. 25	81.0	116	45.3
Ref. 47	85.0	49.5	-
Ref. 62	93.0	9.5	-
Ref. 66	90.0	19.0	-
Ref. 67	85.0	9.2	-
Ref. 68	94.2	27.0	-

Ref. 69	92.0	17.3	-
Ref. 70	91.0	8.9	-
Ref. 71	89.2	14.1	10.0
Ref. 72	88.2	14.9	12.3
Ref. 73	81.0	17.5	27.0
Ref. 74	91.0	52.7	18.0
Ref. 75	88.2	14.9	12.3

Table S4 Comparison of our AgNWs TCFs with other similar kind of work.
